# Acromioclavicular Fracture-Dislocation Fixation Technique With Cerclages and Osteosutures: The “Invisible” Repair

**DOI:** 10.1016/j.eats.2021.12.002

**Published:** 2022-03-16

**Authors:** Gonzalo Samitier, Gustavo Vinagre, David González-Martín

**Affiliations:** a Department of Orthopaedic Surgery and Traumatology, Centro Quirónsalud Aribau, Barcelona, Spain; b Department of Orthopaedic Surgery and Traumatology, Complexo Hospitalar do Médio Ave, Porto, Portugal; c Department of Orthopaedic Surgery and Traumatology, Hospital Lusíadas, Porto, Portugal; d Department of Orthopaedic Surgery and Traumatology, Hospital Universitario de Canarias, Tenerife, Spain; e Universidad de La Laguna, Tenerife, Spain

## Abstract

Surgical treatment usually is indicated for the management of acromioclavicular fracture-dislocations. These are unstable injuries and have shown a high rate of nonunion when managed conservatively. However, surgical strategies often require a second surgery for hardware removal. We describe an arthroscopic-assisted technique to repair the acromioclavicular fracture-dislocation without implants, using a double cerclage and osteosutures. This technique does not require specific instrumentation, avoids clavicle/coracoid drilling, and minimizes secondary irritation related to hardware. This can be used in different anatomic locations and can theoretically reduce the chances of symptomatic hardware, reoperation rates, and iatrogenic fractures.

Distal third clavicle fractures represent between 15% and 25% of all clavicle fractures.^1^^,^^2^ Approximately 25% of these fractures are unstable, also known as acromioclavicular (AC) fracture-dislocations (Fig 1). Anteroposterior stability of the AC joint is provided by the AC ligaments, especially the superior portion with contribution from the joint capsule and deltoid and trapezius muscles.^3^ Vertical stability of the AC joint is provided by the strong coracoclavicular (CC) ligaments. The conoid and trapezoid ligaments originate at the base of the coracoid and insert on the inferior surface of the clavicle from 4 and 2 cm medial to the AC joint, respectively.^4^ In unstable distal clavicle fractures, the proximal fragment is detached from the CC ligaments and displaces superiorly.^5^ Clavicle fractures traditionally have been treated conservatively; however, unstable distal clavicle fractures have been reported to have high nonunion rates when treated nonoperatively,^1^^,^^6^^,^^7^ unlike shaft fractures.^8^

**Fig 1:**
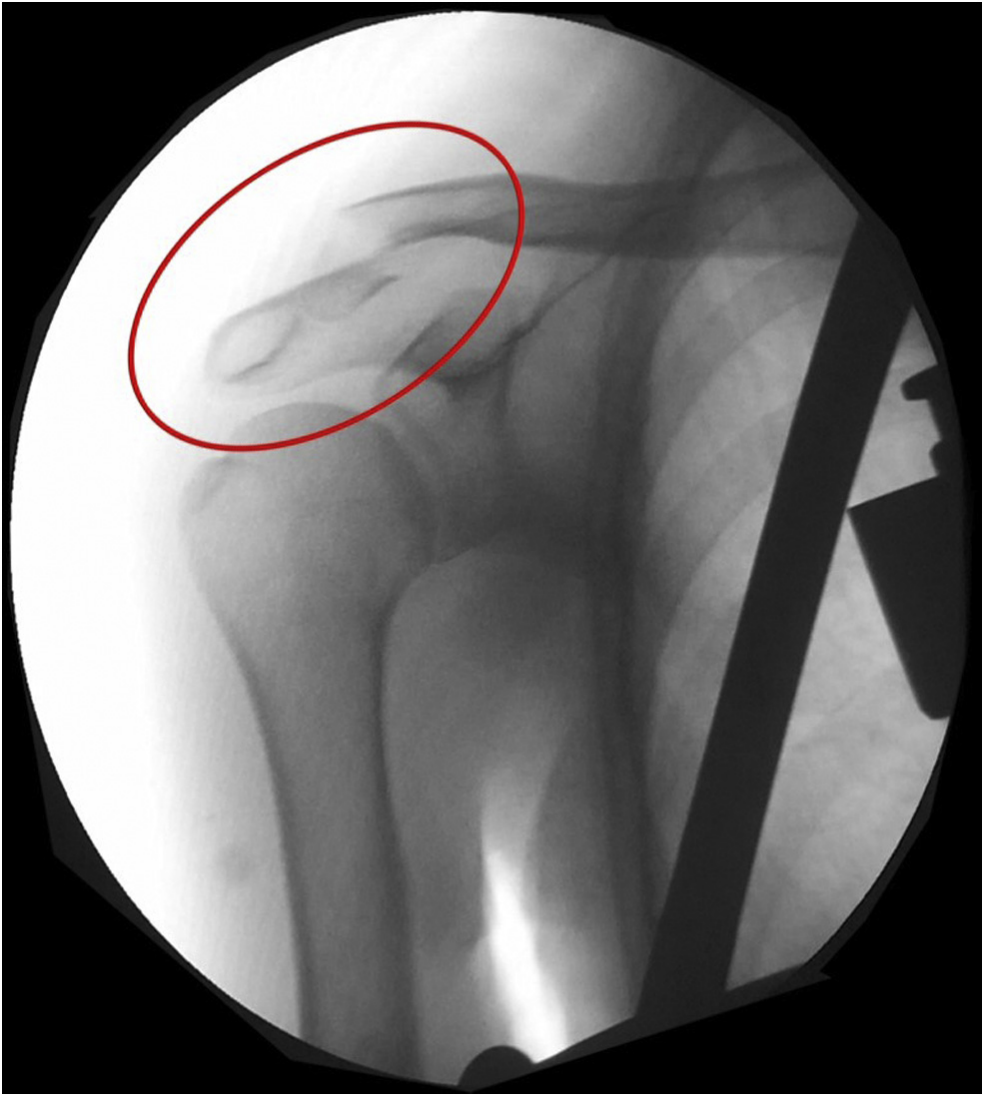
Preoperative radiographic fluoroscopic image of the shoulder (anteroposterior view). Acromioclavicular fracture-dislocation.

While numerous surgical techniques have been described to treat these fractures, most use osteosynthesis plates with either a hook or a locking plate. Despite the excellent union rates,^6^ these techniques are associated with high complication rates, and many of them require a second surgical procedure to remove prominent or painful hardware.^1^ Therefore, minimally invasive arthroscopic techniques with adjustable suture devices have been described in recent years.^9^

The purpose of this Technical Note is to describe the AC fracture-dislocation fixation technique without implants, using a double cerclage and osteosutures. This technique does not require any specific instrumentation, avoids clavicle/coracoid drilling, and minimizes related irritation from hardware (Table 1).

**Table 1:** Advantages and Disadvantages of Description of the AC Fracture-Dislocation Fixation Technique With Cerclages and Osteosutures

Advantages	Disadvantages
• An easy and simplified technique. • Arthroscopically minimally invasive assisted technique. • Better horizontal stability compared with the button technique. • No need for specific instrumentation. • No drilling of the clavicle or the coracoid—avoids iatrogenic fractures. • Minimizes secondary irritation due to the hardware. • Fiber tape suture type cerclages are radiolucent; therefore, there are no concerns of metal mismatch with adjacent implants and no metal debris migration or metallosis. • No need for second hardware removal surgery. • This technique can be used in AC type IV and V dislocations—in these injuries, we recommend adding biological support with autologous semitendinosus graft.	• Arthroscopic skills are required to correctly perform this technique.

AC, acromioclavicular.

## Surgical Technique (With Video Illustration)

**Fig 2:**
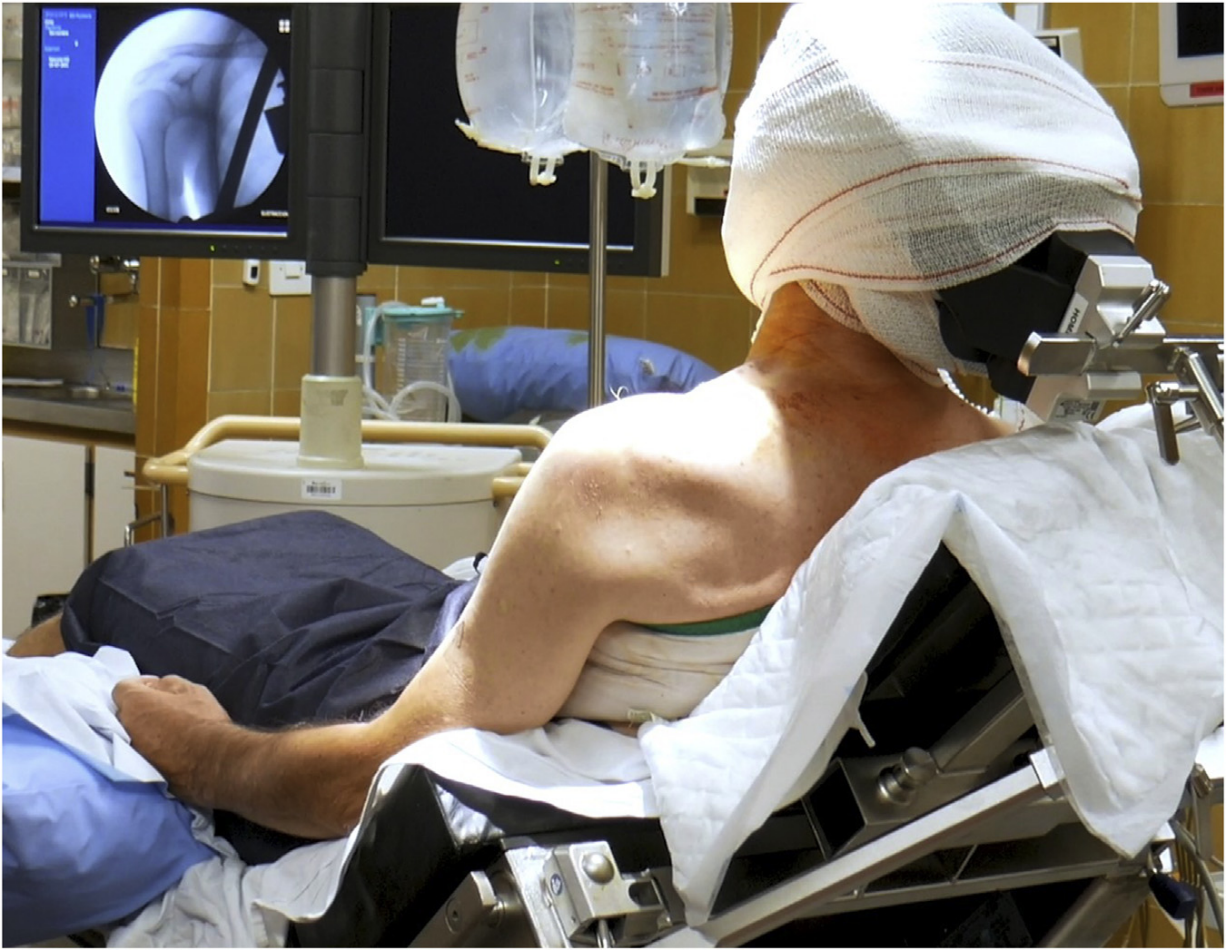
The patient is placed in a beach-chair position. The C-arm is kept on the contralateral side for its possible use during the surgery, specifically to confirm reduction of the acromioclavicular joint. Anatomic landmarks are identified and marked on the skin.

The procedure is performed with the patient under general anaesthesia in the beach-chair position, with the application of traction (3 kg) and under fluoroscopic control (Fig 2; Video 1). Before proceeding with surgery, preoperative fluoroscopy should be used to check and achieve a perfect visualization of the fracture. The C-arm is kept on the contralateral side for its possible use during the surgery, specifically to confirm reduction of AC joint. Anatomic landmarks are identified and marked on the skin.

### Arthroscopy

**Fig 3:**
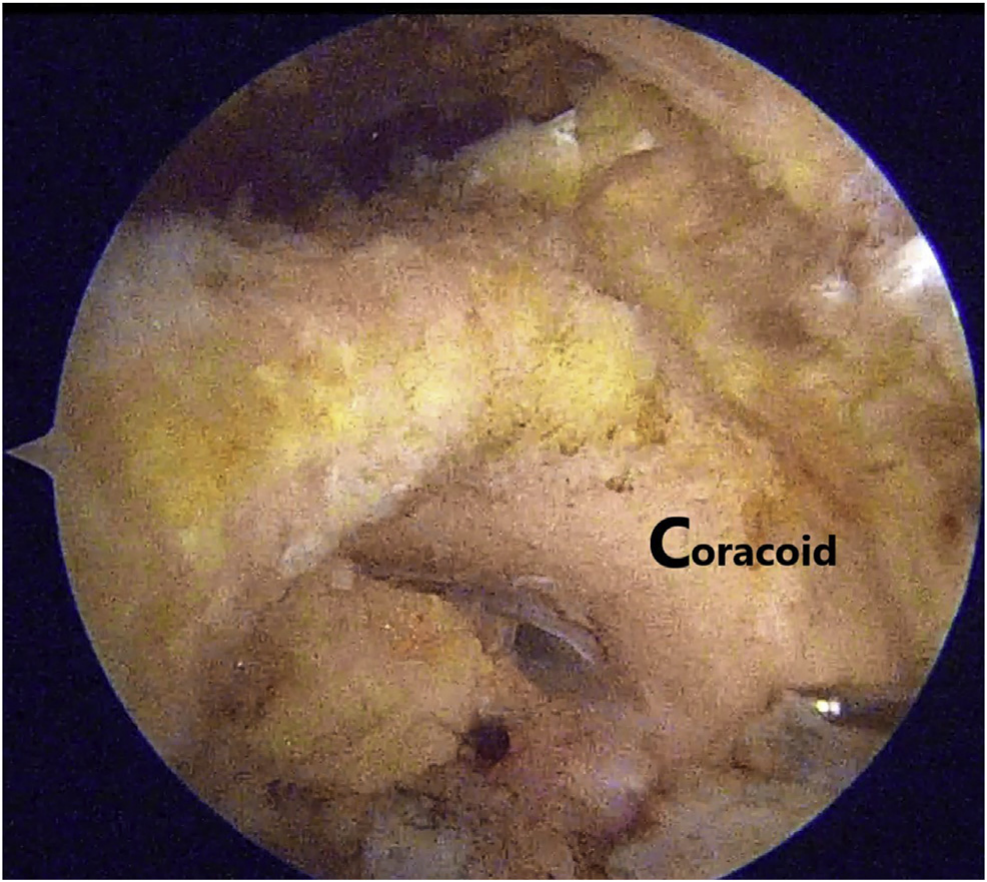
Propper arthroscopic dissection of the base of the coracoid preparation before the repair. Exposure of the coracoid is performed slowly and meticulously using a radiofrequency (RF) ablator with caution during the entire process. The tip of wand can be used to palpate the bony part of the coracoid, and soft tissue is ablated adjacent to the bone only. Once the undersurface of coracoid is identified, with swiping movement of RF ablator, soft tissue from undersurface of the coracoid is removed toward the base.

Through the posterior portal (soft spot 1.5 cm below and medial to the posterolateral corner of acromion), a thorough diagnostic arthroscopy is performed, and an anterior portal is created by outside-in technique to complete the arthroscopy and probe intra-articular structures. Three to four standard portals are used: a standard posterior viewing portal, an anterosuperior instrumental portal—created through the rotator interval—and lateral and/or anterolateral working portals. First, as mentioned previously, diagnostic arthroscopy is initially performed to rule out any associated injuries. With the 30° arthroscope placed in posterior portal and the radiofrequency (RF) ablator through the anterior portal, the rotator interval is opened. The rotator interval is opened carefully from lateral to medial along the superior part of the subscapularis tendon. Exposure of the coracoid is performed slowly and meticulously using a RF ablator with caution during the entire process. The tip of wand can be used to palpate the bony part of the coracoid and soft tissue is ablated adjacent to the bone only. Once the undersurface of coracoid is identified, with swiping movement of RF ablator, soft tissue from undersurface of the coracoid is removed toward the base. Anterior dissection of the coracoid from an anterior portal should be done to section the coracoacromial and coracohumeral ligaments. It is extremely important to have a correct visualization of the base of the coracoid (Fig 3), with care taken to avoid any brachial plexus injury. There is no need to detach the insertion of the pectoralis minor (Table 2).

**Table 2:** Pearls and Pitfalls of the Acromioclavicular Fracture-Dislocation Fixation Technique With Cerclages and Osteosutures

Pearls	Pitfalls
• After patient positioning and before starting the surgery, preoperative fluoroscopy should be used to have a perfect visualization of the fracture. • Diagnostic arthroscopy is initially performed to rule out any associated injuries. • Anterior dissection of the coracoid from anterior portals dissecting coracoacromial and coracohumeral ligaments. • The suture tape cerclages should have a cross-linked configuration, passing under the coracoid and behind the clavicle. • Proper suture cerclage tensioning is given (80-100 Newtons) with a suture tensioning device direct mini-open reduction control, guided by arthroscopic visualization, and fluoroscopic imaging. • Further transosseous sutures (osteosutures) can be placed at the edges of the fracture to reinforce the compression and decrease the chances of fracture site mobility.	• Carefully rotator interval opening should be done from lateral to medial to avoid subscapularis tendon injury. • Avoid to detaching the insertion of pectoralis minor. • Be cautious when dissecting the coracoid, to avoid any brachial plexus injury. • Using different colors (blue and white) for suture tape cerclages is important to avoid suture mismatch and tangling. • Suture knots should be at the anterior or posterior edge of the clavicle to avoid suture skin irritation, pain and the need for a second surgical intervention.

### Mini-Open Approach

**Fig 4:**
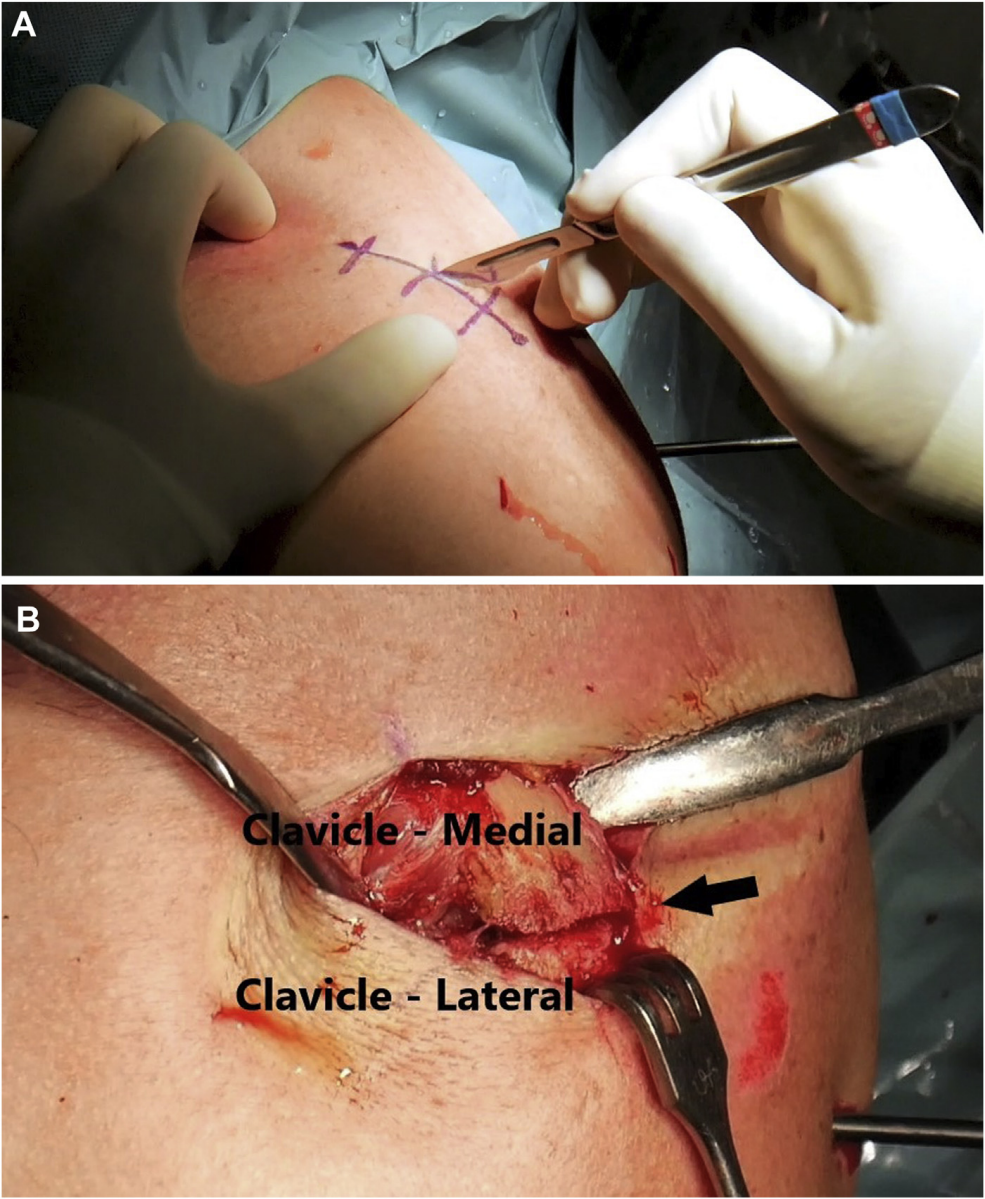
(A) Surgical landmarks and 3- to 4-cm longitudinal skin incision made over the clavicle. (B) Fracture of the distal third of the clavicle (arrow).

A 3- to 4-cm longitudinal incision is made over the clavicle (Fig 4A). Dissection of the fracture site is carried out (Fig 4B). If the time frame between the fracture and the surgery is delayed for some weeks, callus and fibrosis may be present, and it is important to release the entire fragment site for a proper reduction.

### Reduction With Suture Tape Cerclages and Transosseous (Osteosutures) Reinforcement

**Fig 5:**
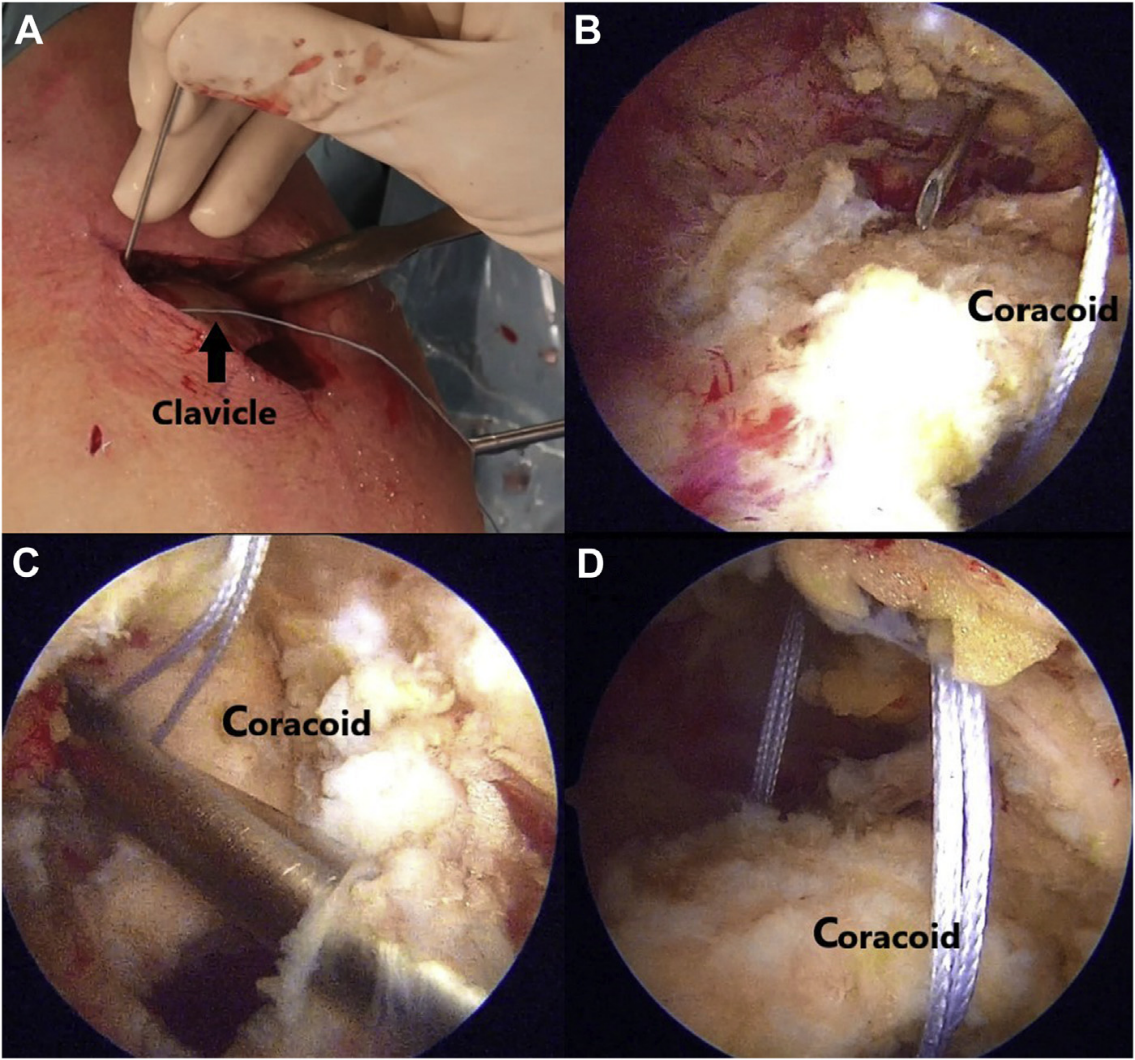
(A) A passing suture is placed with a suture retriever and the position for retrieving the suture is checked with a needle. (B) Arthroscopic view of the position of the needle. (C) The suture is passed under the base of the coracoid with a suture retriever. (D) The suture is shuttled under the base of the coracoid.

**Fig 6:**
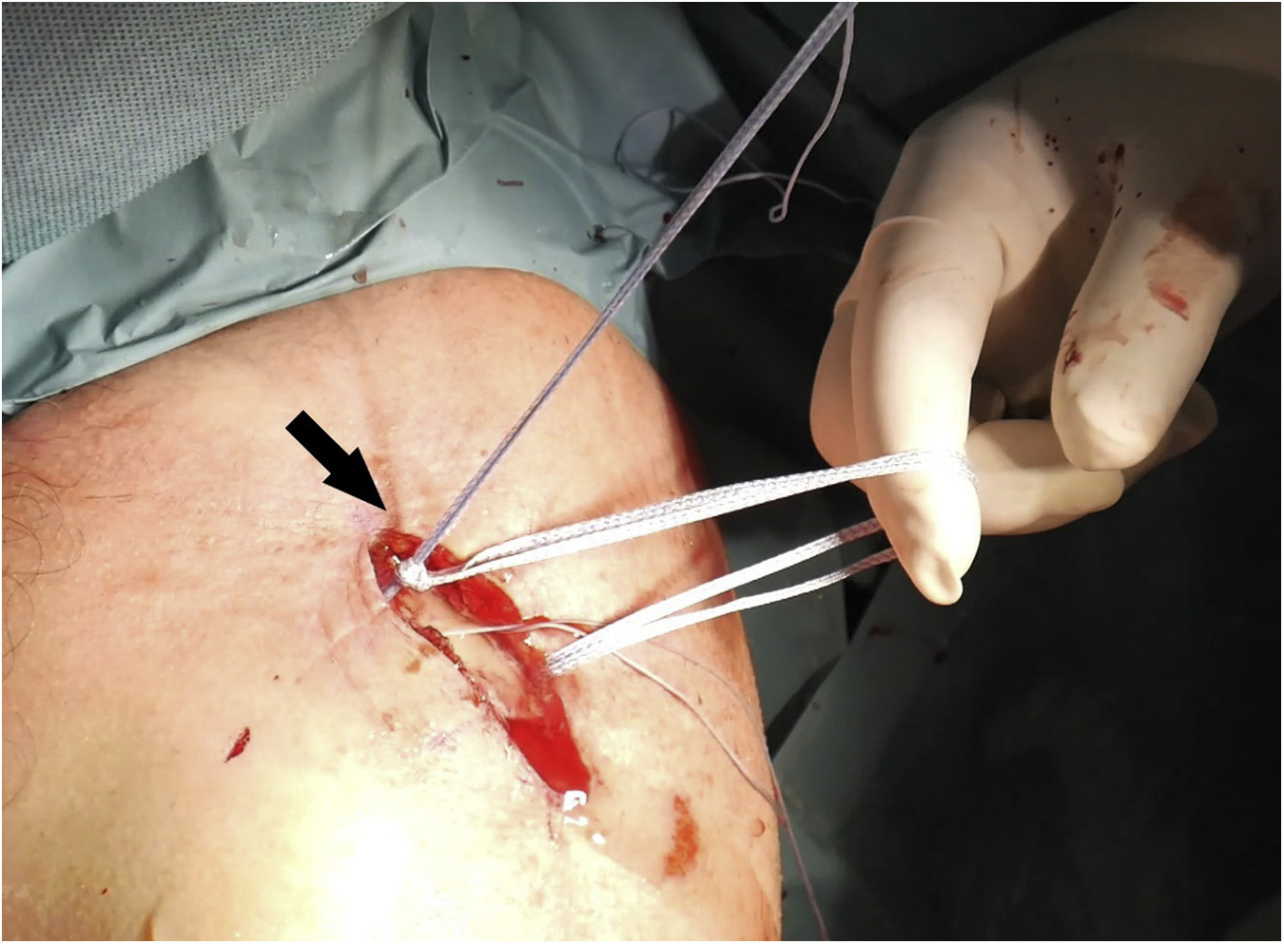
The suture tape cerclage (FiberTape; Arthrex, Naples, FL) is shuttled under the coracoid and behind the medial fragment of the clavicle (arrow).

**Fig 7:**
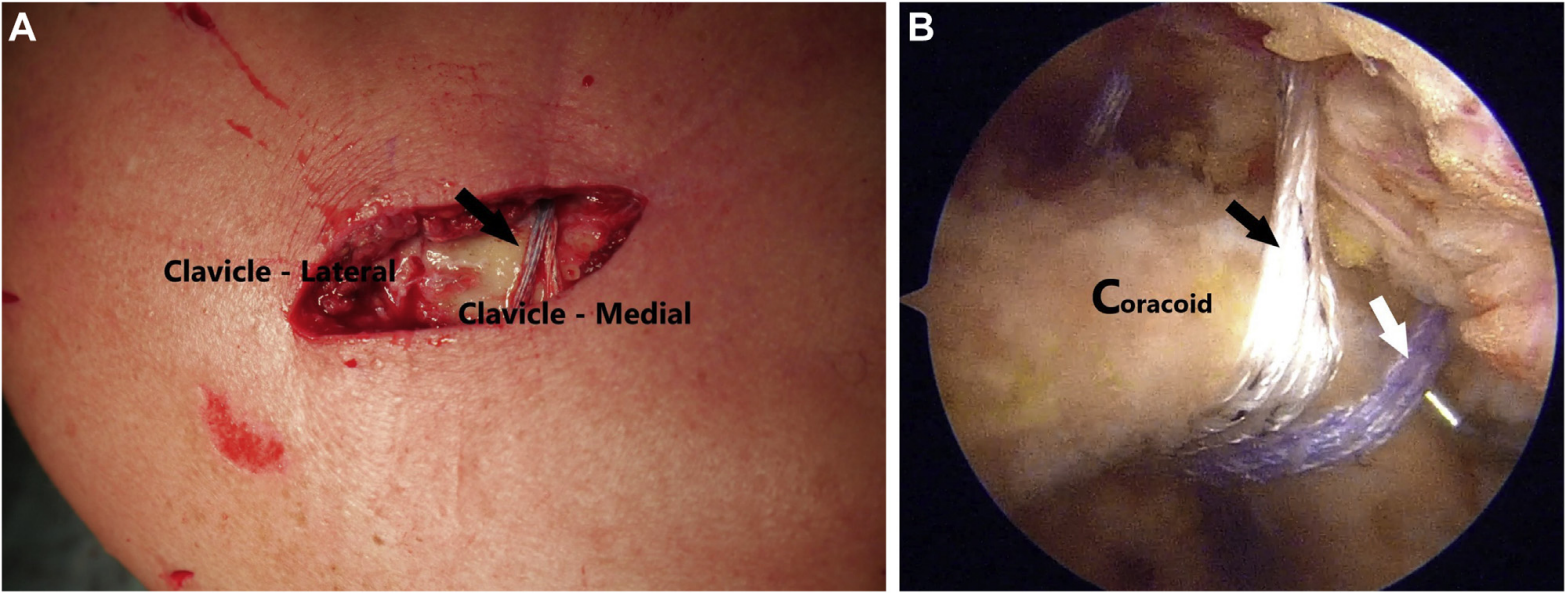
(A) Final cross-linked configuration (arrow) and distal third clavicle fracture reduction. (B) Arthroscopic view—suture tape cerclages passed under the coracoid base. Black arrow: White cerclage mimics the conoid ligament. White arrow: blue cerclage mimics trapezoid ligament.

**Fig 8:**
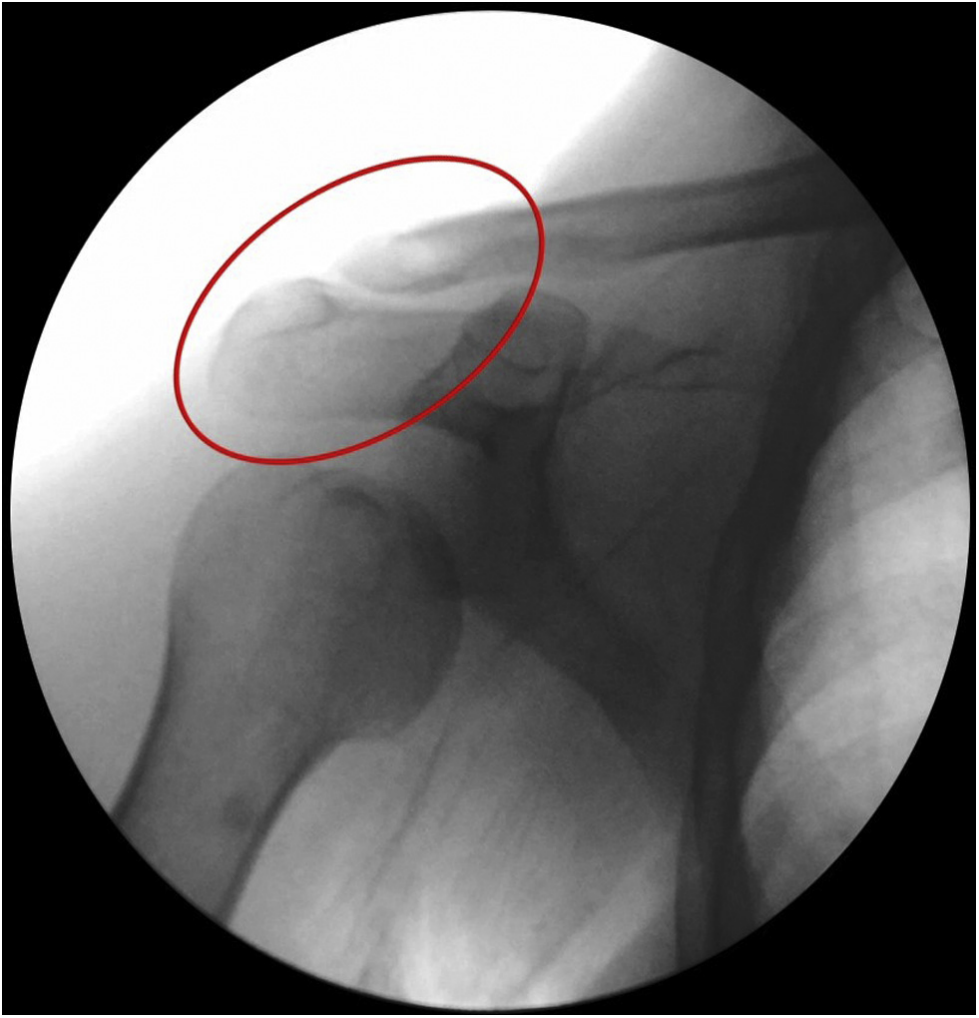
Postoperative fluoroscopic image of the shoulder (anteroposterior view). Acromioclavicular fracture reduction.

A passing suture is placed with a suture retriever (Fig 5) and a suture tape cerclage (FiberTape; Arthrex, Naples, FL) is shuttled under the loop (Fig 6). A second suture tape cerclage is then also shuttled in a cross-linked configuration, passing under the coracoid and behind the medial fragment of the clavicle. Proper tension is given (80-100 Newtons) with a suture tensioning device under direct mini-open reduction control (Fig 7A), guided by arthroscopic visualization (Fig 7B), and fluoroscopic imaging (Fig 8). Further transosseous sutures (osteosutures) can be placed at the edges of the fracture to reinforce the compression and decrease the chances of fracture site mobility.

### Postoperative Rehabilitation

Patients are placed into a standard sling straight after surgery. Passive range of motion and pendulum exercises should be started 1 week postoperatively and proceed over a period of 4 weeks. Afterwards, the sling should be removed and patients start active range of motion and strengthening exercises. Some activities, such as lifting, carrying, pushing, and pulling, should be avoided with the operated ipsilateral upper limb for a period of 2 months after the surgery.

## Discussion

Operative treatment of AC fracture-dislocations remains a challenge. Traditional techniques include locked or hook plates, Kirschner wires, CC screws, suture anchors, or suture tension band wires.^6^^,^^10^, ^11^, ^12^ Despite satisfactory union rates, several complications have been associated with these techniques, including loss of reduction, hardware migration, AC joint arthritis, coracoid fractures, and hardware irritation.^11^^,^^13^ Many of these techniques require a second surgical procedure for hardware removal. Oh et al.,^13^ in a systematic review, have shown a 41% complication rate with the hook plate fixation, which has been considered the gold standard in the treatment of these injuries.

Some of the most commonly used techniques in the literature are the distal clavicle plate,^1^^,^^14^ the hook plate described by Balser in 1976,^15^^,^^16^ and the Dog Bone button.^17^ The first two are open techniques and are frequently associated with complications (infection, pain, no possibility of inspection of possible associated lesions). The distal fragment is often too small to fix with the screws, which increase the chances of repair failure. It can also produce a secondary irritation of the implant to the skin and have the need for a hardware removal second surgery. In addition, plates with vertical implants have been described as perforating and can weaken the coracoid. The Dog Bone button technique also requires drilling the clavicle and the coracoid, it is very technically demanding, and it involves a risk of coracoid or clavicle fracture. It also gives less horizontal stabilization of the fracture with the risk of pseudarthrosis.^1^^,^^18^

The “invisible” repair technique we are described attempts to minimize some of those risks and complications outlined previously. Equivalently to other arthroscopic repair described techniques, there is less soft-tissue damage, resulting in better functional outcomes.^5^^,^^19^, ^20^, ^21^, ^22^, ^23^ In addition, our described technique, is easier and theoretically provides better horizontal stability compared with the button technique. It does not require specific instrumentation, there is less risk of iatrogenic fractures (there is no clavicle or coracoid drilling), and it minimizes the chances of hardware pain and the need for a second surgery for hardware removal.

The same concept of “invisible” repair described in this technique can be used in other anatomic locations such as the humerus, femur, or fibula and also in other injuries such as AC type IV and V dislocations—in these injuries we recommend adding biological support with autologous semitendinosus graft,^23^^,^^24^ even in acute cases, to minimize the risk of failure. In the case of fracture-dislocations, bone healing is the biological mechanism that keeps the injury stabilized in the long term.

In conclusion, the AC fracture-dislocation fixation technique with cerclages and osteosutures is an efficient, reliable, and reproducible technique that potentially improves clinical results and potentially decreases the risk of further complications and failure.

## Supplementary Data

ICMJE author disclosure forms

Video 1 Description of the acromioclavicular fracture-dislocation fixation technique with cerclages and osteosutures: the “invisible” repair.
